# The Impact of the COVID-19 Pandemic on Outcomes in Acute Pancreatitis: A Propensity Score Matched Study Comparing before and during the Pandemic

**DOI:** 10.3390/diagnostics13142446

**Published:** 2023-07-22

**Authors:** Patricia Mihaela Rădulescu, Elena Irina Căluianu, Emil Tiberius Traşcă, Dorin Mercuţ, Ion Georgescu, Eugen Florin Georgescu, Eleonora Daniela Ciupeanu-Călugăru, Maria Filoftea Mercuţ, Răzvan Mercuţ, Vlad Padureanu, Costin Teodor Streba, Cristina Călăraşu, Dumitru Rădulescu

**Affiliations:** 1 UMF Craiova Doctoral School, University of Pharmacy and Medicine Craiova, 200349 Craiova, Romania; paty_miha@yahoo.com; 2 General Surgery Department, University of Medicine and Pharmacy of Craiova, 200349 Craiova, Romania; irina.caluianu@umfcv.ro (E.I.C.); mercutdorin@yahoo.com (D.M.); ion_georgescu@yahoo.com (I.G.); eugenyok@yahoo.com (E.F.G.); dr_radulescu_dumitru@yahoo.com (D.R.); 3 Department of Biology and Environmental Engineering, University of Craiova, 200585 Craiova, Romania; ciupeanudaniela@gmail.com; 4 Department of Ophthalmology, University of Medicine and Pharmacy of Craiova, 200349 Craiova, Romania; nicolcescumaria@gmail.com; 5 Department of Plastic and Reconstructive Surgery, University of Medicine and Pharmacy of Craiova, 200349 Craiova, Romania; 6 Internal Medicine Department, Country Hospital of Craiova, University of Medicine and Pharmacy of Craiova, 200349 Craiova, Romania; 7 Department of Pneumology, University of Pharmacy and Medicine Craiova, 200349 Craiova, Romania; costinstreba@gmail.com (C.T.S.); calarasu.cristina@yahoo.com (C.C.)

**Keywords:** pandemic, COVID-19, acute pancreatitis, MCVL, average crepuscular volume–lymphocyte ratio, IIC, cumulative inflammatory index, NLR, neutrophil–lymphocyte ratio

## Abstract

We aimed to evaluate the outcomes and survival of patients with acute pancreatitis who shared the same clinical form, age, and sex before the pandemic, during the pandemic, and among those with confirmed COVID-19 infection upon hospital admission. This consideration used the sparse data in the existing literature on the influence of the pandemic and COVID-19 infection on patients with acute pancreatitis. To accomplish this, we conducted a multicentric, retrospective case–control study using propensity score matching with a 2:1 match of 28 patients with SARS-CoV-2 infection and acute pancreatitis, with 56 patients with acute pancreatitis pre-pandemic, and 56 patients with acute pancreatitis during the pandemic. The study outcome demonstrated a six-fold relative risk of death in patients with acute pancreatitis and SARS-CoV-2 infection compared to those with acute pancreatitis before the pandemic. Furthermore, restrictive measures implemented during the pandemic period led to a partial delay in the care of patients with acute pancreatitis, which likely resulted in an impairment of their immune state. This, in certain circumstances, resulted in a restriction of surgical treatment indications, leading to a three-fold relative risk of death in patients with acute pancreatitis during the pandemic compared to those with acute pancreatitis before the pandemic.

## 1. Introduction

Acute pancreatitis (AP) is a serious disease characterized by the acute inflammation of the pancreas, necessitating immediate clinical intervention. There is a global increase in AP cases of approximately 34 per 100,000 individuals annually [1]. Predominantly, AP is caused by gallstones and alcohol consumption, though other factors such as hyperlipidemia, hypercalcemia, medication use, infections, post-endoscopic retrograde cholangiopancreatography complications, trauma, HIV, neoplasms, and idiopathic causes (those without a diagnosed origin) are also contributory [2,3,4]. Given the increase in these conditions, a rise in the incidence of AP is anticipated in the forthcoming generation [5]. The clinical course of AP varies, from localized pancreatic inflammation to systemic inflammatory response, categorized according to the revised Atlanta classification into mild AP, denoted by the absence of organ failure and local or systemic complications; moderately severe AP, marked by the presence of transient organ failure or local or systemic complications but the absence of persistent organ failure; and severe AP, characterized by enduring organ failure [6]. Approximately 20% of patients progress to a severe form, potentially leading to systemic inflammatory response syndrome (SIRS) or even multiple organ dysfunction syndromes (MOFS), both of which portend a poor prognosis [7,8]. The global mortality rate associated with AP is approximately 5% but can escalate to 20–30% in patients with severe AP, attributable to the development of pancreatic and extrapancreatic necrosis, subsequent infection, and multiple organ failure (MOF) [8].

In December 2019, a novel coronavirus disease (COVID-19) of unidentified origin emerged in Wuhan, Hubei Province, China [9]. The disease swiftly disseminated to countries globally, imposing a substantial strain on the worldwide healthcare system.

In Romania, the first case of the novel SARS-CoV-2 coronavirus infection responsible for COVID-19 was confirmed on 26 February 2020. The World Health Organization declared the situation a global pandemic on 11 March 2020, and the first death in Romania was reported on 22 March 2020. In the early stage of the pandemic in Romania, the virus’s spread was relatively slow due to the strict measures quickly imposed by authorities. The relaxation of restrictions in the summer of 2020 led to a significant increase in disease spread, causing congestion in hospitals. The COVID-19 pandemic had a significant impact on the healthcare system. In intensive care units, the treatment of COVID-19 patients became increasingly challenging due to the insufficient number of beds and medical personnel, which negatively affected the care of non-COVID-19 patients. Furthermore, the use of some hospitals exclusively as COVID-19 care centers [10] led to a decline in confidence in the medical act, both therapeutically [11] and preventively, such as vaccination [12]. Additionally, resources like hospital and intensive care unit beds, ventilators, and transfusion materials, including protective equipment, were reserved for COVID-19 patients [13]. However, admission rates to intensive care units were higher, suggesting a potential delay in patient presentation to the hospital [14].

The impact of COVID-19 on patients with AP and how it has influenced outcomes and mortality rates is not yet fully understood, as the literature data are limited [15,16,17].

This study aims to compare the outcomes and survival rates of patients with AP of the same severity, both prior to and during the pandemic, as well as patients with AP and confirmed SARS-CoV-2 infection upon hospital admission (Table 1).

## 2. Materials and Methods

We conducted a case–control study involving consecutive cases from two university medical institutions, County Emergency Hospital No. 1 Craiova and “Dr. Stefan Odobleja” Emergency Military Hospital Craiova. This study was carried out after obtaining the approval of the Ethics Committee for each unit, during the period from 1 January 2018 to 30 April 2022.

### 2.1. Inclusion Criteria

The study included all consecutive patients diagnosed with AP, with biological and imaging confirmation in line with the revised Atlanta Criteria [18], from 1 January 2018 to 30 April 2022. These patients were evaluated based on the first blood test conducted prior to the initiation of any drug treatment.

### 2.2. Exclusion Criteria

Patients excluded from the study were those with insufficient hospitalization records in the system, those who had undergone chronic treatment with corticosteroids or immunosuppressive drugs in the last three months, patients with autoimmune diseases, those with incomplete oncological therapy, patients with recurrent episodes of AP, and those diagnosed with chronic pancreatitis.

### 2.3. Study Design

Out of a total of 561 patients with AP hospitalized in the surgery department, the number of patients eligible for the study decreased to 433 after applying the exclusion criteria. We divided the patient cohort into two groups, based on the timeframe before and after the confirmation of the first case of COVID-19 infection in Romania, that is, 26 February 2020. Thus, we defined a pre-COVID-19 group (which includes patients treated between 1 January 2018 and 25 February 2020) and a group during the COVID-19 pandemic (which comprises patients treated between 26 February 2020 and 30 April 2022).

**Figure 1:**
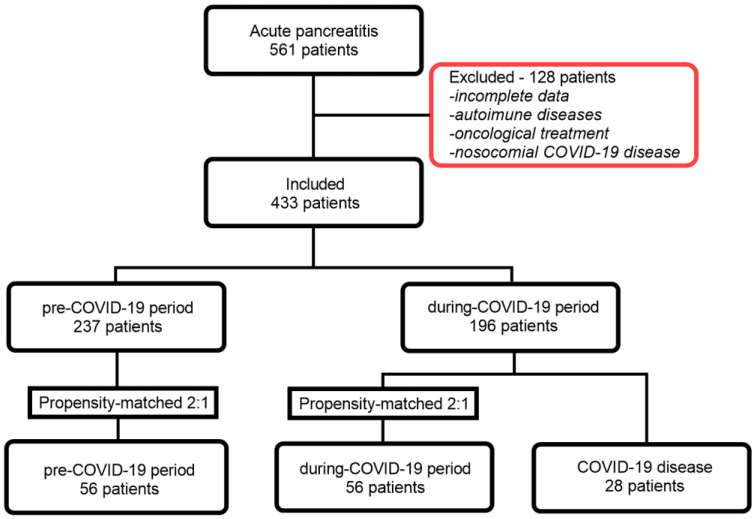
Flow chart of patient inclusion.

Through a propensity score-based approach, we conducted a 1:2 matching of the 28 patients (the AP and COVID-19 group) based on sex, age, and clinical diagnosis, with 56 patients with AP during the pandemic (the during-COVID-19 group) and another 56 patients from the pre-pandemic period (the pre-COVID-19 group). With this method, we obtained three batches of patients (Figure 1). The clinical and paraclinical information of patients with AP were extracted from the informatics system of the two medical institutions. The diagnosis of COVID-19 was confirmed through the reverse transcription polymerase chain reaction (RT-PCR) test conducted on samples obtained by nasopharyngeal swabbing. Only patients with positive RT-PCR tests at the time of admission were included in the study.

### 2.4. Statistical Analysis

Observational studies are often limited by imbalances that may occur between known and unknown confounding factors. To minimize these potential confounding effects in evaluating the rate of complications and mortality before and after the pandemic, as well as among patients infected with SARS-CoV-2, we applied the propensity score matching method [19].

The 28 patients with AP and COVID-19 (*n* = 28) were matched in a 2:1 ratio with patients from the pre-COVID-19 period (*n* = 56) and the during-COVID-19 period (*n* = 56). This was accomplished using a logistic regression model, with the clinical form of AP as the dependent variable and patient characteristics, including sex and age, as covariates. We employed nearest-neighbor matching with a caliper width of 0.2 times the standard deviation of the propensity scores. After completing the matching process, the data were assessed to check for the normal distribution of continuous variables using histograms and the Shapiro–Wilk test. Quantitative variables are expressed as medians (interquartile range) and were analyzed using the Mann–Whitney U test. Categorical variables were presented as frequencies and percentages and were statistically compared using the Chi-square test. A *p*-value of <0.05 was considered statistically significant. All statistical analyses were performed using SPSS software version 26.0 IBM Corporation, Armonk, NY, USA), and the graphs were created using GraphPad Prism v.9.3.0.3.

## 3. Results

In our study, a total of 140 patients (95 males, 67.9%) were evaluated from an initial set of 561 patients with AP admitted to two medical institutions, after applying exclusion criteria and calculating the propensity score. Among them, 28 patients were diagnosed with both SARS-CoV-2 and AP at the time of admission. Using propensity score matching in a 2:1 ratio, we created two control groups, each consisting of 56 patients with AP, one from the pre-COVID-19 period and the other from the COVID-19 pandemic period. We found that parameters such as sex, age, and clinical diagnosis were comparable among the three groups.

### 3.1. Demographic, Clinical and Biological Data of AP Patients from the Pre-COVID-19 vs. during-COVID-19 Group

**Figure 2:**
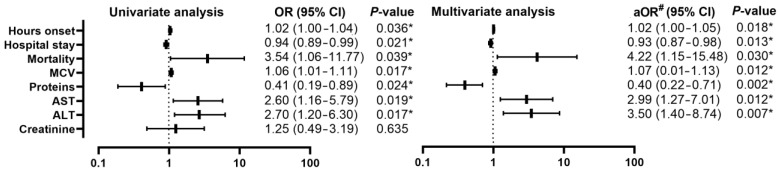
Forest plot with univariate and multivariate analysis of clinical and biological parameters comparing the pre-COVID-19 and during-COVID-19 group; * *p* < 0.05—statistically significant; #—adjusted OR for age, sex, severity; MCV—mean corpuscular volume; AST—aspartate aminotransferase; ALT—alanine aminotransferase.

Comparing patients with AP from the pre-COVID-19 group (*n* = 56) with those from the during-COVID-19 group (*n* = 56) (Table 2), a decrease in the number of days of hospitalization was found [12 (8–19) vs. 9 (7–12); *p* = 0.029], with an OR of 0.94 (95% CI, 0.89–0.99; *p* = 0.021). There is also an increase in the mortality rate from (4/28, 7.1%) to (12/56, 21.4%) (*p* = 0.031) with an OR of 3.54 (95% CI, 1.06–11.77; *p*= 0.039). Statistically significant biological changes were registered among MCV, proteins, AST, and ALT (Table 3), with the maintenance of changes in the univariate analysis (Figure 2), which consisted in the increase in MCV with OR 1.06 (95% CI, 1.01–1.11; *p* = 0.017), protein decrease with OR 0.41 (95% CI, 0.19–0.89; *p* = 0.024), AST increase with OR 2.60 (95% CI, 1.20–6.30; *p* = 0.019), and ALT increase with OR 2.70 (95% CI, 1.20–6.30; *p* = 0.017).

At the multivariate analysis, significant increases in the OR value were observed among mortality 4.22 (95% CI, 1.15–15.48) and among ALT 3.50 (95% CI, 1.40–8.74), the rest of the parameters having slightly increased OR values.

### 3.2. Demographic and Biological Data of Pre-COVID-19 AP Patients vs. COVID-19 AP Patients

**Figure 3:**
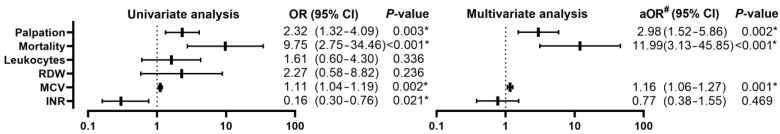
Forest plot with univariate and multivariate analysis of clinical and biological parameters comparing the pre-COVID-19 group and the AP group with COVID-19; * *p* < 0.05—statistically significant; #—adjusted OR for age, sex, severity; RDW—red cell distribution width; MCV—mean corpuscular volume; INR—international normalized ratio.

Comparing the patients with AP from the pre-COVID-19 group (*n* = 56) with those from the group with AP with COVID-19 (*n* = 28), a worsening was found from the point of view of the clinical examination by decreasing the number of patients who presented only pain, from (30/56, 53.6%) to (7/28, 25.0%) (*p* = 0.008), with an OR of 2.3 (95% CI, 1.32–4.09; *p* = 0.003). The death rate increased from (4/56, 7.1%) to (12/28, 42.9%) (*p* = 0.028), with an OR of 9.75 (95% CI, 2.75–34.66; *p* < 0.001) (Figure 3).

The biological parameters with statistically significant differences were leukocytes, RDW, MCV, creatinine, of which only MCV increase with OR 1.11 (95% CI, 1.04–1.19; *p* = 0.002) and INR increase with OR 0.16 (95% CI, 0.30–0.76; *p* = 0.021).

Multivariate analysis after adjusting for age, sex and severity showed a slight increase in mortality with an OR 11.99 (95% CI, 3.13–45.85; *p* < 0.001), palpation sensitivity with an OR 2.98 (95% CI, 1.52–5.86; *p* < 0.001). *p* = 0.002), without significant change in OR value of MCV 1.16 (95% CI, 1.06–1.27; *p* = 0.001).

### 3.3. Demographic and Biological Data of AP Patients in the during-COVID-19 Period vs. AP Patients with COVID-19

**Figure 4:**
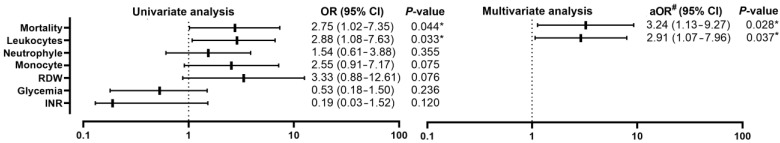
Forest plot with univariate and multivariate analysis of clinical and biological parameters comparing the during-COVID-19 group and the AP group with COVID-19; * *p* < 0.05—statistically significant; #—adjusted OR for age, sex, severity; RDW—red cell distribution width; INR—international normalized ratio.

By comparing patients with AP from the during-COVID-19 group (*n* = 56) with those from the group with AP and COVID-19 (*n* = 28), the mortality rate increased from (12/56, 21.4%) to (12/28, 42.9%) (*p* = 0.028) with an OR 2.75 (95% CI, 1.02–7.35; *p* = 0.044). Statistically significant differences were recorded in the biological parameters of leukocytes, neutrophils, monocytes, RDW, blood glucose, and INR, with only the increase in leukocytes having significance in the univariate analysis (Figure 4) with an OR of 2.88 (95% CI, 1.08–7.63; *p* = 0.033).

Multivariate analysis showed a slight increase in the OR to 3.24 (95% CI, 1.13–9.27; *p* = 0.028) for mortality, without significant change in the value of the OR of leukocytes 2.91 (95% CI, 1.07–7.96; *p* = 0.037).

### 3.4. Comparison of Patients Discharged Alive and Those Who Died in the Pre-COVID-19 Group

**Figure 5:**
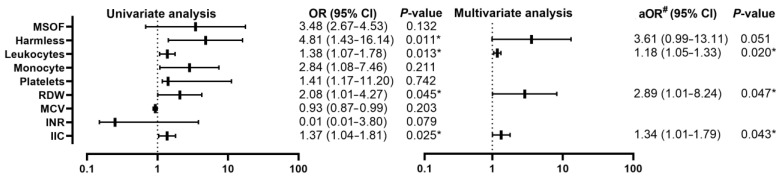
Forest plot with univariate and multivariate analysis of clinical and biological parameters comparing mortality in the pre-COVID-19 group; * *p* < 0.05—statistically significant; #—adjusted OR for age, sex, severity; MSOF—Multiple organ failure; RDW—red cell distribution width; MCV—mean corpuscular volume; INR—international normalized ratio; IIC—cumulative inflammatory index.

By comparing living AP patients (*n* = 52) with those who died (*n* = 4) from the pre-COVID-19 group (Table 4), multiple organ dysfunction was found in three patients from the deceased group (3/4, 75%) and in six of the living group (6/52, 11.5%) (*p* = 0.001), but without significance in the univariate analysis, the Harmless Acute Pancreatitis Score (HAPS) had higher values among the deceased, thus two patients (2/4, 50%) had a score of “1” and two patients (2/4, 50%) had a score of “3”, with a statistically significant difference *p* < 0005, with an OR of 4.81 (95% CI, 1.43–16.14; *p* = 0.011). From the biological point of view (Table 5), statistically significant differences were recorded in leukocytes, monocytes, platelets, RDW, MCV, INR, and IIC, but the univariate analysis (Figure 5) showed significance only in the increase in leukocytes with an OR of 1.38 (95% CI, 1.07–1.78; *p* = 0.013), increased RDW with an OR of 2.08 (95% CI, 1.01–4.27; *p* = 0.045) and increased IIC with an OR of 1.37 (95% CI, 1.04–1.81; *p* =0.025).

The multivariate analysis did not confirm the efficiency of the HAPS, but with the increase in OR among RDW 2.89 (95% CI, 1.01–8.27; *p* = 0.047), maintaining a similar value of OR among IIC of 1.34 (95% CI, 1.01–1.79; *p* = 0.043) and slightly decreased OR among leukocytes of 1.18 (95% CI, 1.05–1.33; *p* = 0.020).

### 3.5. Comparison of Patients Discharged Alive and Those Who Died in the during-COVID-19 Group

**Figure 6:**
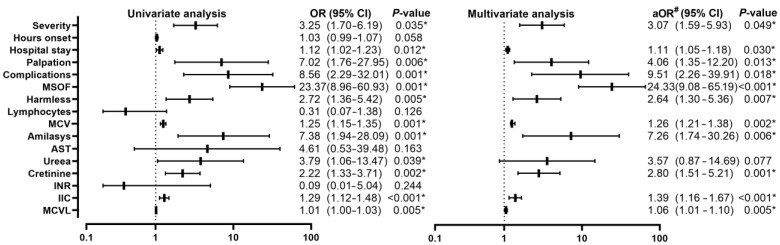
Forest plot with univariate and multivariate analysis of clinical and biological parameters comparing mortality in the during-COVID-19 group; * *p* < 0.05—statistically significant; #—adjusted OR for age, sex, severity; MSOF—Multiple organ failure; MCV—mean corpuscular volume; AST—aspartate aminotransferase; INR—international normalized ratio; NLR—neutrophil-lymphocyte ratio; IIC—cumulative inflammatory index; MCVL—lymphocyte MCV ratio.

Comparing patients with AP alive (*n* = 44) with those who died (*n* = 12) from the during-COVID-19 group, it was observed that (11/12, 91.7%) had the severe form compared to the group of patients alive, in that we encountered all three forms of pancreatitis with the predominance of the moderately severe (25/44, 56.8%) and severe (13/44, 29.5%) forms, with a statistically significant difference *p* = 0.001, with an OR of 3.25 (95% CI, 1.70–6.19; *p* = 0.035). The number of hours since the onset of symptoms did not register significance in the univariate analysis. An increase in hospitalization days was found among deceased patients [9 (6–10) vs. 12.5 (9–25); *p* = 0.004], with an OR of 1.12 (95% CI, 1.02–1.23; *p* = 0.012). From the point of view of the clinical examination, from the group of those who died, 2 patients presented abdominal tenderness (2/12, 16.7%) and 10 patients showed muscle defense (10/12, 83.3%), compared to those alive who had presented pain (2/44, 45.5%), tenderness (11/44, 25.0%), and muscle defense (13/44, 29.5%), with a statistically significant difference *p* = 0.002, with an OR of 7.02 (95% CI, 1.76–27.95; *p* = 0.006). Complications were present in 7 (7/44, 15.9%) of the living patients, compared to those who died, of which 10 (10/12, 83.3%), with a statistically significant difference *p* < 0.001, with an OR of 8.56 (95% CI, 2.29–32.01; *p* = 0.001). MSOF was found in 10 of the deceased patients (10/12, 83.3%), in comparison with the living patients, only 2 of them had MSOF (2/44, 4.5%), with a highly statistically significant difference *p* < 0.001, with an OR of 23.37 (95% CI, 8.96–60.93; *p* = 0.001). HAPS had higher values among deceased patients compared to the group of living patients who had lower values, with an OR of 2.72 (95% CI, 1.36–5.42; *p* = 0.005). Regarding the biological parameters, statistically significant differences were recorded in lymphocytes, MCV, amylase, AST, urea, creatinine, INR, IIC, and MCVL, with insignificant difference among leukocytes in the univariate analysis, but with significance in increased MCV with an OR of 1.25 (95% CI, 1.15–1.35; *p* = 0.001), increased amylase with an OR of 7.38 (95% CI, 1.94–28.09; *p* = 0.001), AST without significant difference on univariate analysis, increased urea with an OR of 3.79 (95% CI, 1.06–13.47; *p* = 0.039), increased creatinine with an OR of 2.22 (95% CI, 1.33–3.71; *p* = 0.002), INR without statistical significance in univariate analysis, IIC increased with an OR of 1.29 (95% CI, 1.12–1.48; *p* < 0.001), and increased MCVL with an OR of 1.01 (95% CI, 1.00–1.03; *p* = 0.005) (Figure 6).

Multivariate analysis showed significant results among severity, with an OR of 3.07 (95% CI, 1.59–5.93; *p* = 0.049), duration of hospitalization with an OR of 1.11 (95% CI, 1.05–1.18; *p* = 0.030), palpation with an OR of 4.06 (95% CI, 1.35–12.20; *p* = 0.013), complications with an OR of 24.33 (95% CI, 9.08–65.19; *p* < 0.001), HAPS with an OR of 2.64 (95% CI, 1.30–5.36; *p* = 0.007). Among the biological parameters, increased VEM remained significant with an OR of 1.26 (95% CI, 1.21–1.38; *p* = 0.002), increased amylase with an OR of 7.26 (95% CI, 1.74–30.26; *p* = 0.006), creatinine increased with an OR of 2.80 (95% CI, 1.51–5.21; *p* = 0.001), IIC increased with an OR of 1.39 (95% CI, 1.16–1.67; *p* < 0.001) and MCVL increased with an OR of 1.06 (95% CI, 1.01–1.10; *p* = 0.005).

### 3.6. Comparison of Patients with AP and COVID-19 Disease Discharged Alive and Those Who Died

**Figure 7:**
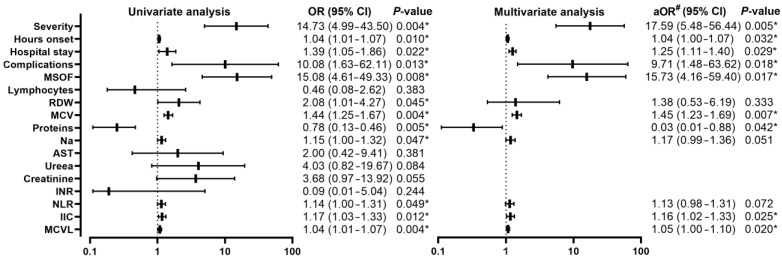
Forest plot with univariate and multivariate analysis of clinical and biological parameters comparing mortality in the group of patients with AP and COVID-19 disease; * *p* < 0.05—statistically significant; #—adjusted OR for age, sex, severity; RDW—red cell distribution width; MCV—mean corpuscular volume; Na—sodium; AST—aspartate aminotransferase; NLR—neutrophil lymphocyte ratio; IIC—cumulative inflammatory index; MCVL—lymphocyte MCV ratio.

Comparing patients with AP alive (*n* = 16) with those who died (*n* = 12) in the during-COVID-19 group, it was observed that all patients who died had the severe form (12/12, 100%) compared to the group of patients alive in which we encountered only patients with pancreatitis with a predominance of mild (3/16, 18.8%) and moderately severe (13/16, 81.2%) forms, with a statistically highly significant difference *p* < 0.001, with an OR of 14.73 (95% CI, 4.99–43.50; *p* = 0.004). Regarding the hours since the onset of symptoms, a higher median was found among deceased patients [12 (12–24) vs. 71.5 (39–72); *p* = 0.001], with an OR of 1.04 (95% CI, 1.01–1.07; *p* = 0.010). An increase in the number of days of hospitalization among deceased patients was also observed [8 (7–10) vs. 15 (14–32); *p* = 0.001], with an OR of 1.39 (95% CI, 1.05–1.86; *p* = 0.0.022). From a clinical point of view, palpation found an absence of patients who only presented pain on abdominal palpation among those who died, this was also present in a proportion of 43.8% (7/16) among those who survived. Regarding abdominal sensitivity, it was absent among those who survived, and the muscular defense had a proportion of 56.2% (9/16), compared to those who died, who only showed sensitivity (6/12, 50%) and muscle defense (6/12, 50%), with a statistically significant difference (*p* = 0.001), but without significance in the univariate analysis. MSOF was present in 66.7% (8/12) of deceased patients and in 2.5% (2/16) of living patients, with a statistically significant difference (*p* < 0.001), with an OR of 15.08 (95% CI, 4.61–49.33; *p* = 0.008). The biological parameters showed statistically significant differences in lymphocytes, RDW, MCV, proteins, Na, ALT, urea, creatinine, INR, NLR, IIC, and MCVL. The univariate analysis (Figure 7) showed a significant increase in RDW with an OR of 2.08 (95% CI, 1.01–4.27; *p* = 0.045), an increase in MCV with an OR of 1.44 (95% CI, 1.25–1.67; *p* =0.004), protein decrease with an OR of 3.18 (95% CI, 1.31–7.73; *p* = 0.011), Na increase with an OR of 1.15 (95% CI, 1.00–1.32; *p* = 0.047), NLR increase with a OR of 1.14 (95% CI, 1.03–1.33; *p* = 0.049), IIC increase with an OR of 1.17 (95% CI, 1.03–1.33; *p* = 0.012), and MCVL with an OR of 1.04 (95% CI, 1.01–1.07; *p* = 0.004).

Multivariate analysis showed similar OR values, the highest values being among severity with 17.59 (95% CI, 5.48–56.44; *p* = 0.005), complications with an OR of 9.71 (95% CI, 1.48–63.62; *p* = 0.018), and MSOF with an OR of 15.73 (95% CI, 4.16–59.40; *p* = 0.017). Among biological parameters, the multivariate analysis showed significance among RDW with an OR of 1.38 (95% CI, 0.53–6.19; *p* = 0.333), MCV with an OR of 1.45 (95% CI, 1.23–1.69; *p* = 0.007), proteins with an OR of 4.12 (95% CI, 1.26–13.47; *p* = 0.019), IIC with an OR of 1.16 (95% CI, 1.02–1.33; *p* = 0.025), and MCVL with an OR of 1.05 (95% CI, 1.00–1.10; *p* = 0.020).

## 4. Discussion

This study aligns with other research examining the impact of COVID-19 infection on the outcomes of patients with AP [15,16,20], demonstrating an increased mortality rate among patients with AP and COVID-19 disease. Additionally, to our knowledge, this study is the first to highlight the direct impact of the pandemic on the evolution of patients with AP, as well as those with COVID-19 disease and AP, by calculating the risk of death for each patient group separately.

The COVID-19 pandemic has had significant consequences on public health measures [17], disrupting people’s daily lives and reducing access to medical services, leading to changes in the number of hospital visits for other conditions. Hospitals have reported an unexplained decrease in hospitalizations for serious medical conditions, such as myocardial infarction and other coronary diseases [21,22], as well as for abdominal surgical conditions [23], including acute appendicitis [24], diverticulitis [25], and acute cholecystitis [26]. The results of these studies coincide with those of our research, which highlighted a decrease in the number of AP cases treated in the two centers, from 237 in the pre-pandemic period to 196 during the pandemic, over the same period. This is justified by the tendency of patients to delay or avoid medical care, behavior that can lead to a deterioration of their health status and potentially increase mortality [27,28].

It was found that a higher percentage of patients with AP and COVID-19 come from rural areas. This is partly due to the lower rate of mask-wearing in public spaces in these regions compared to urban areas [29,30]. In addition, rural residents often have various comorbidities that can increase their susceptibility to more severe forms of COVID-19 or AP [31]. Socio-economic factors, such as poverty and lack of education, can enhance their vulnerability to SARS-CoV-2 infection and potentially lead to a higher mortality rate [32].

During the pandemic, people were encouraged to take a conservative approach to less specific symptoms of AP, such as abdominal pain, vomiting, diarrhea, headaches, and pharyngalgia [33,34,35], to see if pulmonary symptoms develop in the following days before seeking medical consultation, in order to prevent the spread of COVID-19 infection.

According to a study by Tabiei et al. [36] on self-medication, antibiotics are the most commonly used drugs after analgesics, which could explain the partial relief of symptoms and the delay in presenting to health services. This finding is consistent with the results of our study, in which we observed an increase in the number of hours from symptom onset during the pandemic compared to the pre-COVID-19 period.

In the context of the COVID-19 pandemic, it was found that patients who died had a longer period of time from symptom onset to presentation at healthcare services. This trend was identified both among patients with AP and patients diagnosed with AP and COVID-19 simultaneously. According to some studies, the duration between the onset of AP symptoms and presentation for medical care can have a decisive impact on the severity of the disease [37]. Similarly, delaying medical assistance in the case of COVID-19 can lead to exacerbation of symptoms and complications, with the potential of increasing the mortality rate [38]. Thus, these data underscore the vital importance of early medical intervention in the effective management of COVID-19 and AP.

The severity of AP is positively correlated with serum levels of ALT and AST [39,40], and this was also highlighted in our study, where we observed significantly increased values of these liver enzymes in patients with AP during the pandemic compared to the pre-pandemic period. It is important to note that AST and ALT can also be increased in the case of non-steroidal anti-inflammatory drug use [41], which could be a situation encountered during the pandemic when patients resorted to self-medication [42]. Brooks et al. examined the psychosocial impact of measures during the COVID-19 pandemic and found an increase in stress, anxiety, and frustration levels [43], factors that, according to several studies, are positively associated with increased transaminase levels [44,45,46]. The pandemic led to lifestyle changes, and some people resorted to increasing alcohol consumption [47,48,49] as a stress management mechanism or to occupy free time, which could have led to an increase in AST and ALT levels [50,51]. Limited access to medical care and proper monitoring during the pandemic led to delays in diagnosing or managing pre-existing liver conditions, which could have led to an increase in AST and ALT levels [52]. A study by Harper et al. showed that elevated ALT levels are associated with early mortality in patients with AP [53]. Another study by Tenner et al. suggested that both elevated AST and ALT are associated with an increased risk of mortality in patients with AP [54].

The harmful effects of delayed treatment have been observed in the past following natural disasters [55,56]. It was anticipated that patients during the pandemic would avoid hospital visits and emergency medical care due to fear of infection with the SARS-CoV-2 virus, leading to increased morbidity and mortality in patients with chronic and acute diseases [57,58,59], including AP, due to a lack of awareness of their severity. Additional measures to prevent SARS-CoV-2 infection, including PCR testing and additional chest imaging examinations, also contributed to the delayed presentation of patients with AP to the hospital, as evidenced in our study by the increased time from the onset of symptoms to hospital presentation, leading to delayed medical assistance. Delayed initiation of fluid resuscitation and other supportive treatments can worsen clinical outcomes of patients with severe AP [60], leading to tissue hypoperfusion and hypoxia, which can increase the complexity and severity of the disease, as evidenced in our study by the increased severity of cases during the pandemic.

The increase in severity of AP cases resulted in a higher rate of MSOF during the pandemic, with a doubling of the rate in patients with AP and COVID-19 disease, a finding also made by Hadi et al. [61], leading to a more severe course with an increased mortality rate.

According to data from our study, during the pandemic, we recorded an increased incidence of Multiple Organ Dysfunction Syndrome (MODS), which seems to be determined by an increase in the number of cases of severe AP during this period. Petrov and his collaborators found that COVID-19 can also contribute to the development of MODS, either through direct organ damage induced by the virus or through a systemic inflammatory response called a “cytokine storm” [62]. In our study, we found that patients with AP and COVID-19 had a MODS rate twice as high compared to patients suffering from AP in the pre-pandemic period. Therefore, it seems that patients with AP and COVID-19 may have a significantly higher risk of developing MODS, considering the individual contribution of each disease to this syndrome. We assume that this increase in the incidence of MODS led to a more severe course of AP and an increasing mortality rate.

In the context of the surgical treatment of patients with AP, there was a decrease in the number of patients who underwent surgical interventions for infected necrosis during the pandemic, especially in those who also had SARS-CoV-2 infection. This decrease may be attributed to the impact of the pandemic on the restrictions imposed on surgical services, such as limiting the number of surgeries, rescheduling certain surgical interventions and prioritizing medical resources towards patients with COVID-19 [63,64,65]. Another scenario could be that patients arrived at the hospital in an advanced stage of the disease, making surgical intervention unfeasible due to multiorgan failure that can occur if rehydration treatment is initiated late [66], ultimately leading to the death of these patients.

The hematological changes identified in our study following SARS-CoV-2 infection included leukocytosis. According to a study by Lippi et al., leukocytosis was introduced as a hematological parameter used to predict the severity of COVID-19 disease. This hematological change may reflect the patient’s evolution towards more severe and disappointing clinical conditions [67].

In our study, we did not include other predictive serum markers, such as C-reactive protein, procalcitonin, interleukin-6, and interleukin-8, or the CD64 index of neutrophils. The reason is that these markers cannot accurately predict the prognosis and severity of AP [68,69]. Also, in our study, these markers were not widely used.

The Neutrophil-to-Lymphocyte Ratio (NLR) is an inflammatory marker used to estimate prognosis in various conditions such as infectious diseases, cardiovascular diseases, tumors, autoimmune diseases, and surgical complications [70,71,72,73]. NLR has also been studied in the context of AP [68,74,75]. In our research, we found that NLR is only significant in predicting the mortality of AP patients infected with the SARS-CoV-2 virus. According to studies, a high NLR level upon admission of COVID-19 patients can anticipate a more severe disease course, including death. This is explained by the fact that a high NLR level suggests the presence of a severe inflammatory reaction, which can cause tissue damage and organ dysfunction [76].

The cumulative inflammatory index (IIC) is a new inflammatory marker that can be calculated by multiplying MCV (mean corpuscular volume) with RDW (red cell distribution width) and neutrophils, all divided by the absolute number of lymphocytes multiplied by 1000 [77]. The IIC value reflects changes in the white blood cell lineage, such as neutrophils and lymphocytes, combined with the effects of inflammation on the red blood cell lineage, reflected by the MCV and RDW values. In our study, the IIC showed significance in predicting mortality in AP patients, both pre-pandemic, during the pandemic, and in patients with AP and SARS-CoV-2 infection.

The MCV to lymphocyte ratio (MCVL), recognized for its ability to predict complications in patients with AP [77], was only significant in terms of predicting mortality in AP patients infected with SARS-CoV-2.

In our study, we observed an increase in the Mean Corpuscular Volume (MCV) in patients diagnosed with AP during the pandemic compared to those diagnosed with the same condition pre-pandemic. We also identified increased MCV values in patients with AP who died during the pandemic, regardless of their SARS-CoV-2 infection status. This increase could be correlated with an intensification of alcohol consumption during this period [47,78]. Studies conducted by Toh and colleagues highlight that alcohol abuse can lead not only to elevated MCV but also to the onset of AP, thereby establishing an indirect link between elevated MCV and the occurrence of AP [79].

This increase in MCV could be associated with erythrocyte metabolism and homeostasis, which are related to the body’s overall antioxidant properties. Alteration of the erythrocyte membrane can lead to a reduced antioxidant capacity [80,81]. Increased MCV might be the result of a potential compromise in the antioxidant capacity of macrocytic erythrocytes, which, independent of inflammation, may lead to endothelial dysfunction [82]. Endothelial dysfunction is present in patients with severe AP, and the degree of endothelial impairment may be associated with disease severity, potentially leading to increased mortality [83].

Initially, it was believed that the SARS-CoV-2 virus primarily affects the respiratory system, but subsequent reports have highlighted the spread of the virus to multiple organs, especially those expressing the ACE-2 receptor, including gastrointestinal (GI) epithelial cells. Elements of the SARS-CoV-2 virus have been detected in stool samples from infected patients. Among the organs expressing the ACE-2 receptor are the pancreas, which has receptors in ductal cells, acinar cells, and islet cells [65].

In addition to the pathophysiological changes that occur in AP, it is speculated that the effect of SARS-CoV-2 infection promotes the leakage of pancreatic lipase into visceral adipose tissue by affecting adipocytes and pancreatic cells. This process can lead to the production of unsaturated fatty acids and lipotoxic organ failure, likely caused by the cytokine storm that follows infection. In the case of severe AP and severe SARS-CoV-2 infection, a remarkable similarity in increased levels of cytokines, such as IL-1, IL-6, IL-8, and IL-10, has been observed. However, in severe AP, only the level of IL-1α is increased, while IL-1β shows low values [84]. In SARS-CoV-2 infection, regardless of infection severity, both IL-1α and IL-1β have high values [85], contributing to triggering cytokine storms and uncontrolled immune responses [86]. These aspects are involved in the pathogenesis of severe pneumonia, sepsis, and shock [87]. Combining the cytokine storm from COVID-19 disease with that from AP can create a cumulative effect on the body, increasing the risk of multiorgan dysfunction and, consequently, mortality.

According to our data, a decrease in the initial number of lymphocytes was observed in patients suffering from AP and COVID-19 disease who did not survive, which is in line with other studies [88,89]. These studies focused solely on COVID-19 disease and highlighted several factors that may contribute to lymphopenia in SARS-CoV-2 infection, including the presence of the ACE-2 receptor on the surface of lymphocytes [90]. This allows these cells to be infected, which subsequently leads to their lysis. The cytokine storm induced by COVID-19 may promote apoptosis or necrosis of lymphocytes, leading to their depletion [91], suggesting that the virus can affect and consume immune cells, inhibiting cell-mediated immune responses [92]. Another study conducted by Lippi et al. [70], which analyzed the lymphocyte count in patients with different forms of COVID-19 disease (mild, severe, and critical), showed a low lymphocyte count in all three groups, with no statistically significant differences between them. Also, in a study conducted by Guan et al. on 1099 patients, it was found that lymphopenia was present in 83.2% of patients at the time of admission, and severe disease was characterized by more pronounced lymphopenia compared to those with less severe forms of the disease [93].

The higher mortality among patients with AP during the pandemic can be partially explained by the unintended impact of pandemic measures on the population [94]. These measures included social distancing, reduced outdoor activities, and increased indoor time. One hypothesis is related to changes in diet as a result of the pandemic period, which affected access and availability of fresh food and led to changes in shopping habits [95].

In stressful situations, people tend to consume tastier but less healthy foods [96]. The COVID-19 pandemic has led to increased stress levels and, consequently, unhealthy changes in eating habits [97]. There are several hypotheses regarding the mechanisms by which a diet poor in fruits and vegetables and rich in processed meat can contribute to inflammation through reactive oxygen and nitrogen species, influencing the pathogenesis of AP [98].

Diet also impacts the composition of the gut microbiome, potentially inducing a pro-inflammatory state, increasing the risk of AP, and potentially worsening the clinical course of the disease [99,100]. This is similar to findings from our study, where we observed an increase in mortality during the COVID-19 pandemic period compared to the period before the pandemic.

A Dutch study showed that changes in eating behavior during the pandemic, such as skipping hot meals, could contribute to malnutrition [42]. Furthermore, it has been demonstrated that COVID-19 can increase the incidence of malnutrition even in communities with the lowest risk of malnutrition, suggesting that COVID-19 disease or associated complications can trigger physiological processes that can affect the immune system, increase the length of hospital stay, and mortality [101,102,103]. This is consistent with the findings of our study, where we observed a decrease in serum protein levels and an increase in the duration of hospital stay in patients with AP and SARS-CoV-2 infection who died.

**Figure 8:**
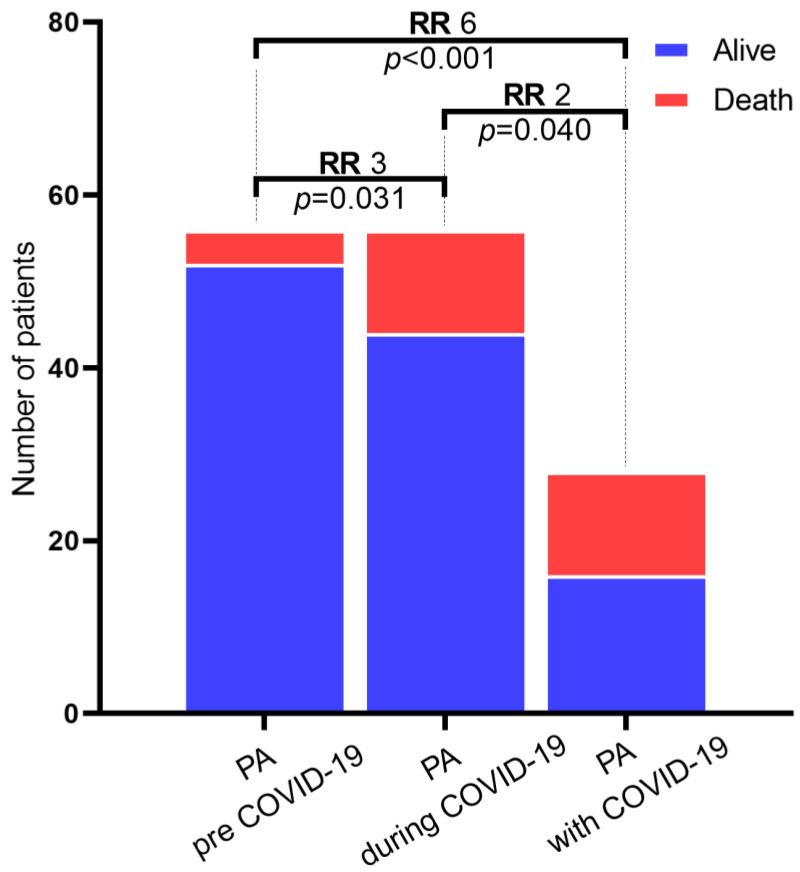
The relative risk of death of patients with AP by comparison between the pre-COVID-19 and during-COVID-19, during-COVID-19 and AP with COVID-19, and pre-COVID-19 and AP with COVID-19 period.

This study revealed the negative impact of the pandemic and SARS-CoV-2 infection on patients with AP, and this result was highlighted by calculating the relative risk (RR) for each individual period compared to the period before the pandemic (Table 6). The evidence from the study shows that patients during the pandemic had a relative death risk of three compared to patients with AP before the pandemic, underlining the negative impact of the pandemic on patients during this period (Figure 8). In addition, patients with AP and SARS-CoV-2 infection had a relative death risk of six compared to patients with AP before the pandemic.

### Study Limitation

The main limitation of this study is its retrospective nature. Propensity score matching can only control observed covariates, such as sex, age, and the severity of AP (AP). However, there may be unobserved covariates that were not adjusted to balance the baseline characteristics between the groups exposed and unexposed to the pandemic and the SARS-CoV-2 virus, which could lead to selection bias. Also, a large number of subjects were excluded after propensity score matching due to the limited number of patients with AP and SARS-CoV-2 infection, even despite using a complete matching algorithm. External sources could not be excluded either, such as pre-hospital treatment, which could have influenced the inflammatory state of patients at the time of blood collection. Further large cohort studies are needed to generalize the results obtained in this study.

## 5. Conclusions

According to our study results, the SARS-CoV-2 infection had a significant impact on patients with AP, increasing the risk of mortality six-fold. Surprisingly, the pandemic itself, through the implementation of drastic measures, caused a partial delay in patient care, either due to the fear of going to the hospital, due to difficulties in admitting patients to the hospital for epidemiological reasons or even due to the limited capacity of intensive care units. These factors likely altered the immune status of patients with AP, having a negative impact on survival and leading to a three-fold increase in the risk of mortality compared to the pre-pandemic period. Our study generated several hypotheses regarding the cause of increased mortality in patients with AP during the COVID-19 pandemic, which can be considered in future research to prepare the medical system for similar situations created by a pandemic.

## Figures and Tables

**Table 1:** Research questions.

Primay Results	Secondary Results
- Was there a delay in hospital admission for patients with AP during the pandemic? - How did the implementation of COVID-19 infection prevention measures influence the timing and type of treatment administered to patients with AP? - Did the COVID-19 pandemic have an impact on the clinical outcomes and survival rate of patients with AP?	Is there an increased risk of death for patients with AP during the COVID-19 pandemic and, if so, can this be quantified? Did COVID-19 infection have an impact on the clinical outcomes and survival rate of patients with AP? Can COVID-19 infection exacerbate the risk of death in patients with AP and, if so, can this be numerically evaluated?

**Table 2:** Comparison of demographic and clinical data of patients in the pre-pandemic AP group (a) during the pandemic (b) and in those with AP with COVID-19 disease (c).

Variable	AP Pre-COVID-19 ^a^ (*n* = 56)	AP during-COVID-19 ^b^ (*n* = 56)	AP with COVID-19 ^c^ (*n* = 28)	*p* ^a,b^	*p* ^a–c^	*p* ^b,c^
Area				0.705 †	0.277 †	0.434 †
Urban	27 (8.2%)	25 (46.6%)	10 (35.7%)			
Rural	29 (51.8%)	31 (55.4%)	18 (64.3%)			
Age	49 (38–63)	50 (44–64)	49 (41–63)	0.305	0.887	0.475
Sex				1 †	1 †	1 †
M	38 (67.9%)	38 (67.9%)	19 (67.9%)			
W	18 (32.1%)	18 (32.1%)	9 (32.1%)			
AP Severity				1 †	1 †	1 †
Mild	6 (10.7%)	6 (10.7%)	3 (10.7%)			
Moderately_s	26 (46.4%)	26 (46.4%)	13 (46.4%)			
Severe	24 (42.9%)	24 (42.9%)	12 (42.9%)			
Hours onset	24 (12–35)	36 (24–48)	24 (12–72)	0.002 *	0.161	0.785
Hospital stay	12 (8–19)	9 (7–12)	9 (7–15)	0.029 *	0.110	0.696
Palpation				0.064 †	0.008 *†	0.462 †
Pain	30 (53.6%)	20 (35.7%)	7 (25.0%)			
Tenderness	14 (25.0%)	13 (23.2%)	6 (21.4%)			
Guarding	12 (21.4%)	23 (41.1%)	15 (53.6%)			
HTN				0.403 †	0.306 †	0.685 †
Yes	21 (37.5)	13 (23.2%)	6 (21.4%)			
No	35 (62.5%)	43 (76.8%)	22 (78.6%)			
Diabetes				0.809 †	0.848 †	0.723 †
Yes	11 (19.6%)	10 (17.9%)	6 (21.4%)			
No	45 (80.4%)	46 (82.1%)	22 (78.6%)			
Complications				0.547 †	0.746 †	0.774 †
Yes	20 (32.15%)	17 (30.4%)	9 (32.1%)			
No	36 (64.3%)	39 (69.6%)	19 (67.9%)			
MSOF				0.468 †	0.179 †	0.380 †
Yes	8 (14.3%)	12 (21.4%)	8 (28.6%)			
No	48 (85.7%)	44 (78.6%)	20 (71.4%)			
HAPS				0.541 †	0.317 †	0.642 †
0	26 (46.4%)	21 (37.5%)	8 (28.6%)			
1	22 (39.3%)	23 (41.1%)	12 (42.9%)			
2	5 (8.9%)	5 (8.9%)	5 (17.9%)			
3	3 (5.4%)	7 (12.5%)	3 (10.7%)			
Surgery				0.508 †	0.264 †	0.514 †
Yes	6 (10.7%)	4 (7.1%)	1 (3.6%)			
No	50 (89.3%)	52 (92.9%)	27 (96.4%)			
Mortality				0.031 *†	<0.001 *†	0.028 *†
Yes	4 (7.1%)	12 (21.4%)	12 (42.9%)			
No	52 (92.9%)	44 (78.6%)	16 (57.1%)			

* *p* < 0.05—statistically significant; † Chi-square test; HTN—high blood pressure; MSOF—Multiple system organ failure; Moderately_s—moderately severe. HAPS—Harmless Acute Pancreatitis Score.

**Table 3:** Comparison of the biological data of patients in the group with AP before the pandemic (a), during the pandemic (b) and those with AP with COVID-19 disease (c).

Variable	AP Pre-COVID-19 ^a^ (*n* = 56)	AP during-COVID-19 ^b^ (*n* = 56)	AP with COVID-19 ^c^ (*n* = 28)	*p* ^a,b^	*p* ^a–c^	*p* ^b,c^
Leucocytes (×10^3^/µL)	11.9 (7.2–16.3)	9.2 (7.6–13.3)	12.6 (9.4–19.8)	0.192	0.044 *	0.002 *
Neutrophile (×10^3^/µL)	8.2 (11.9–13.8)	8.0 (5.5–10.2)	9.1 (6.5–16.1)	0.658	0.133	0.021 *
Lymphocyte (×10^3^/µL)	1.5 (0.9–2.0)	1.4 (1.0–2.0)	2 (1.0–2.5)	0.658	0.220	0.074
Monocyte (×10^3^/µL)	0.8 (0.5–1.2)	0.6 (0.5–0.8)	0.9 (0.5–1.4)	0.085	0.287	0.015 *
Platelets (×10^3^/µL)	195.9 (176.4–271.5)	183.0 (148.0–284.8)	237.0 (189.0–253.0)	0.254	0.453	0.323
Hb (g/dL)	13.6 (12.1–14.5)	12.5 (11.7–14.5)	14.0 (10.8–15.9)	0.073	0.790	0.414
Ht (%)	38.6 (36.4–44.6)	38.2 (34.5–43.6)	35.6 (33.3–43.4)	0.159	0.178	0.857
RDW	12.8 (11.1–14.4)	13.2 (12.4–14.4)	14 (13.7–14.7)	0.311	0.001 *	0.004 *
MCV (fL)	88.5 (85.2–95.8)	93.6 (88.7–99.0)	95.6 (88.4–102.6)	0.002 *	0.002 *	0.362
Proteins (g/dL)	6.5 (6.0–7.2)	6.0 (5.4–7.0)	6.4 (5.1–7.3)	0.001 *	0.342	0.543
Amylase (U/L)	320 (81.3–495.5)	248.0 (172.0–515.0)	323.5 (186.0–1114.3)	1	0.288	0.384
Na (mmol/L)	134 (129.0–140.0)	137.6 (131.0–140.0)	136.5 (132.0–140.0)	0.218	0.565	0.610
K (mmol/L)	4.5 (3.8–5.0)	3.9 (3.7–4.8)	4.1 (3.5–4.8)	0.064	0.207	0.917
Glycemia (mg/dL)	136 (85.0–158.0)	111 (95.0–212.0)	99 (93.0–134.8)	0.691	0.102	0.035 *
AST (U/L)	42 (26.3–110.0)	88 (37.5–184.0)	58.0 (33.0–117.0)	0.019 *	0.314	0.333
ALT (U/L)	48.5 (20.0–172.0)	87 (52.5–226.5)	58.0 (24.0–159.0)	0.001 *	0.393	0.144
Urea (mg/dL)	33 (30.0–49.0)	32 (24.3–69.0)	35.5 (26.0–59.0)	0.550	0.932	0.930
Creatinine (mg/dL)	0.9 (0.7–1.1)	0.7 (0.67–1.0)	0.7 (0.6–1.4)	0.022 *	0.305	0.887
INR	1.1 (1.1–1.3)	1.2 (1.0–1.4)	1.0 (1.0–1.1)	0.554	0.000 *	0.002 *
NLR	6.5 (3.0–12.9)	5.8 (3.4–12.2)	5.3 (3.3–11.2)	0.852	0.776	0.955
IIC	7.7 (3.2–15.7)	7.6 (3.9–17.4)	9.9 (4.2–18.7)	0.376	0.352	0.556
MCVL	63.6 (41.5–98.8)	61.2 (46.3–105.5)	43.4 (36.9–101.3)	0.306	0.494	0.051

* *p* < 0.05—statistically significant; Hb—hemoglobin; Ht—hematocrit; RDW—red cell distribution width; MCV—mean corpuscular volume; Na—sodium; K—potassium; AST—aspartate aminotransferase; ALT—alanine aminotransferase; INR—international normalized ratio; NLR—neutrophil lymphocyte ratio; IIC—cumulative inflammatory index; MCVL—lymphocyte MCV ratio.

**Table 4:** Comparison of demographics of patients alive and those who died in the groups with AP pre-COVID-19, during-COVID-19, and in those with AP and COVID-19 disease.

	AP Pre-COVID-19			AP during-COVID-19			AP with COVID-19		
Variable	Alive (*n* = 52)	Deceased (*n* = 4)	*p*	Alive (*n* = 44)	Deceased (*n* = 12)	*p*	Alive (*n* = 16)	Deceased (*n* = 12)	*p*
Area			0.266 †			0.282 †			0.069 †
Urban	24 (46.2%)	3 (75%)		18 (40.9%)	7 (58.3%)		8 (50%)	1 (8.3%)	
Rural	48 (53.8%)	1 (25%)		26 (59.1%)	5 (41.7%)		8 (50%)	11 (91.7%)	
Age	46.5 (35–61)	57 (51–63)	0.251	47 (44–62)	61 (44–64)	0.179	45.50 (37–61)	57.5 (42–77)	0.377
Sex			0.153 †			0.382 †			0.129 †
M	34 (65.4%)	4 (100%)		29 (65.9%)	9 (75%)		9 (56.2%)	10 (83.3%)	
W	18 (34.6%)	0		15 (34.1%)	3 (25%)		7 (43.8%)	2 (16.7%)	
AP Severity			0.057 †			0.001 *†			0.000 *†
Mild	6 (11.5%)	0		6 (13.6%)	0		3 (18.8%)	0	
Moderately_s	26 (50%)	0		25 (56.8%)	1 (8.3%)		13 (81.2%)	0	
Severe	20 (38.5%)	4 (100%)		13 (29.5%)	11 (91.7%)		0	12 (100%)	
Hours onset	24 (12–30)	39 (6.00–72)	0.844	24 (24–48)	48 (36–66)	0.004 *	12 (12–24)	71.5 (39–72)	0.002 *
Hospital stay	12 (8–19)	13.50 (5–22)	0.798	9 (6–10)	12.5 (9–25)	0.004 *	8 (7–10)	15 (14–32)	0.003 *
Palpation			0.081 †			0.002 *†			0.001 *†
Pain	30 (57.7%)	0		20 (45.5%)	0		7 (43.8%)	0	
Tenderness	12 (23.1%)	2 (50%)		11 (25.0%)	2 (16.7%)		0	6 (50%)	
Guarding	10 (19.2%)	2 (50%)		13 (29.5%)	10 (83.3%)		9 (56.2%)	6 (50%)	
HTN			0.751 †			1 †			0.184 †
Yes	17 (32.7%)	1 (25%)		11 (25.0%)	3 (25.0%)		2 (12.5%)	4 (33.3%)	
No	35 (67.3%)	3 (75%)		33 (75.0%)	9 (75.0%)		14 (87.5%)	8 (66.7%)	
Diabetes			0.113 †			0.903 †			0.690 †
Yes	9 (17.3%)	2 (50%)		8 (18.2%)	2 (16.7%)		3 (18.8%)	3 (25%)	
No	43 (82.7%)	2 (50%)		36 (81.8%)	10 (83.3%)		13 (81.2%)	9 (75%)	
Complications			0.536 †			<0.001 *†			0.005 *†
Yes	18 (34.6%)	2 (50%)		7 (15.9%)	10 (83.3%)		2 (12.5%)	8 (66.7%)	
No	34 (65.4%)	2 (50%)		37 (84.1%)	2 (16.7%)		14 (87.5%)	4 (33.3%)	
MSOF			0.001 *†			0.000 *†			<0.001 *†
Yes	6 (11.5%)	3 (75%)		2 (4.5%)	10 (83.3%)		2 (12.5%)	8 (66.7%)	
No	46 (88.5%)	1 (25%)		42 (95.5%)	2 (16.7%)		14 (87.5%)	4 (33.3%)	
HAPS			0.000 *†			0.020 *†			0.103 †
0	26 (50%)	0		20 (45.5%)	1 (8.3%)		7 (43.8%)	1 (8.3%)	
1	20 (38.5%)	2 (50%)		18 (40.9%)	5 (41.7%)		5 (31.2%)	7 (58.4%)	
2	5 (9.6%)	0		3 (6.8%)	2 (16.7%)		1 (6.2%)	4 (33.3%)	
3	1 (1.9%)	2 (50%)		3 (6.8%)	4 (33.3%)		3 (18.8%)	0	
Surgery			0.472 †			0.278 †			0.240 †
Yes	6 (11.5%)	0		4 (9.1%)	0		0	1 (8.3%)	
No	46 (88.5%)	4 (100%)		40 (90.9%)	12 (100%)		16 (100%)	11 (91.7%)	

* p < 0.05—statistically significant; † Chi-square test; moderately_s—moderately severe. HAPS—Harmless Acute Pancreatitis Score.

**Table 5:** Comparison of biological parameters of living patients and those who died in the groups with pre-COVID-19 AP, during-COVID-19 and in those with AP and COVID-19 disease.

	AP Pre-COVID-19			AP during-COVID-19			AP with COVID-19		
Variable	Alive N (%)	Deceased N (%)	*p*	Alive N (%)	Deceased N (%)	*p*	Alive N (%)	Deceased N (%)	*p*
Leucocyte (×10^3^/µL)	11.7 (7.2–16.3)	21.4 (16.5–26.0)	0.005 *	9.2 (7.5–13.3)	7.8 (7.1–19.9)	0.952	16.4 (10.2–19.8)	10.7 (7.7–31.1)	0.456
Neutrophil (×10^3^/µL)	7.0 (5.2–12.9)	18.5 (14.0–22.9)	0.005 *	8.2 (4.5–10.2)	6.1 (5.7–17.6)	0.535	9.1 (6.7–16.1)	9.2 (5.7–27.1)	0.544
Lymfocyte (×10^3^/µL)	1.6 (0.9–2.2)	1.1 (1.0–1.1)	0.372	1.6 (1.1–2.0)	1.0 (0.7–1.0)	0.000 *	2.4 (2.0–16.1)	1.1 (0.9–1.3)	0.018 *
Monocyte (×10^3^/µL)	0.7 (0.5–1.1)	1.5 (1.5–1.6)	0.006 *	0.6 (0.4–0.8)	0.7 (0.5–1.2)	0.810	1.0 (0.6–1.7)	0.8 (0.3–1.3)	0.124
Platelet (×10^3^/µL)	193.6 (170.7–222.8)	377 (362.0–391.3)	0.005 *	191.0 (148.0–284.0)	148.0 (95.5–299.4)	0.105	237.0 (189.8–287.2)	207.0 (51.9–249.3)	0.167
Hb (g/dL)	13.7 (12.2–14.5)	12.4 (11.3–13.4)	0.143	12.4 (11.7–14.3)	9.0 (8.5–17.1)	0.222	14.0 (11.2–16.0)	13.2 (10.1–15.6)	0.208
Ht (%)	38.6 (36.9–45.0)	37.1 (34.3–39.8)	0.339	38.6 (35.6–43.3)	26.4 (25.7–44.3)	0.271	35.6 (34.2–43.4)	38.7 (29.5–44.2)	0.327
RDW	12.8 (11.9–13.7)	14.7 (13.9–23.8)	0.022 *	12.5 (11.7–14.3)	14.9 (12.4–15.6)	0.254	13.8 (13.7–14.0)	14.7 (14.2–15.2)	0.004 *
MCV	89.1 (85.4–96.3)	82.4 (79.9–84.9)	0.011 *	89.9 (88.3–97.3)	105.0 (100.1–107.2)	0.000 *	90.3 (88.1–93.8)	102.6 (98.6–102.8)	0.000 *
Proteins (g/dL)	6.6 (6.1–7.2)	6.1 (5.7–6.5)	0.097	6.0 (5.5–7.3)	6.3 (5.4–5.6)	0.457	6.9 (6.4–7.4)	5.1 (4.5–5.12)	0.000 *
Amylase (U/L)	319 (80.5–492.5)	480.5 (456.0–503.9)	0.161	213.5 (112.8–414.5)	515.0 (344.0–1196.5)	0.002 *	192.0 (186.0–1294.0)	342.0 (215.0–1144.3)	0.816
Na (mmol/L)	137.0 (129.0–140.0)	129.5 (125.0–133.8)	0.222	138.0 (135.2–142.5)	131.0 (128.0–135.0)	0.056	137.0 (135.0–141.5)	131.0 (123.0–139.0)	0.022 *
K (mmol/L)	4.5 (3.8–5.0)	5.1 (4.0–6.1)	0.315	3.9 (3.7–4.5)	4.8 (3.5–5.2)	0.475	3.9 (3.8–4.2)	4.2 (3.4–6.9)	0.133
Glycemia (mg/dL)	136 (84.0–158.0)	125.5 (95.0–125.5)	1	104.0 (94.3–189.0)	203.0 (111.0–465.0)	0.027	99.0 (93.0–129.3)	99.0 (86.8–150.5)	0.675
AST (U/L)	42.5 (28.8–119.0)	29.5 (21.0–37.6)	0.056	66.0 (36.0–169.0)	105.0 (95.0–906.0)	0.043 *	40.0 (27.0–102.3)	90.0 (40.0–372.0)	0.015 *
ALT (U/L)	48.5 (19.0–172.0)	42.5 (33.0–51.6)	0.949	87.0 (33.0–230.0)	58.0 (57.5–26.0)	0.327	44.0 (24.0–277.0)	99.0 (14.3–159.0)	0.576
Urea (mg/dL)	33.0 (30.0–49.0)	78.5 (26.0–128.6)	0.610	32.0 (18.5–57.0)	69.0 (30.0–125.5)	0.004 *	31.0 (18.0–52.0)	59.0 (29.5–182.0)	0.004 *
Creatinine (mg/dL)	0.9 (0.7–1.0)	2.8 (2.7–4.7)	0.702	0.7 (0.6–0.9)	4.2 (0.7–6.1)	0.002 *	0.7 (0.6–1.2)	1.7 (0.7–3.3)	0.032 *
INR	1.1 (1.1–1.2)	6.1 (1.4–10.5)	0.003 *	1.1 (0.1–1.4)	1.4 (0.4–1.6)	0.001 *	1.0 (0.9–1.0)	1.1 (1.0–1.2)	0.000 *
NLR	6.3 (2.6–11.5)	16.6 (13–21.0)	0.007 *	5.1 (2.2–8.5)	16.4 (6.6–18.0)	0.000 *	3.7 (2.6–3.9)	8.8 (6.9–18.0)	0.001 *
IIC	6.5 (2.8–13.9)	21.7 (19.3–23.9)	0.003 *	6.7 (2.6–14.4)	22.2 (14.6–27.2)	0.000 *	4.6 (36.7–43.4)	13.9 (11.7–21.3)	0.002 *
MCVL	55.9 (39.9–100.6)	77.2 (74.6–79.8)	0.445	54.8 (45.4–80.7)	106.9 (98.6–245.1)	0.000 *	37.7 (36.7–43.4)	96.7 (74.7–112.8)	0.001 *

* *p* < 0.05—statistically significant; Hb—hemoglobin; Ht—hematocrit; RDW—red cell distribution width; MCV—mean corpuscular volume; Na—sodium; K—potassium; AST—aspartate aminotransferase; ALT—alanine aminotransferase; INR—international normalized ratio; NLR—neutrophil lymphocyte ratio; IIC—cumulative inflammatory index; MCVL—lymphocyte MCV ratio.

**Table 6:** The Chi-Square test and the relative risk (RR) through the comparison of the death of AP patients between the pre-COVID-19 and during-COVID-19 period, during-COVID-19 and AP with COVID-19, and pre-COVID-19 and AP patients with COVID-19.

Compared Groups	Odd Ratio CI 95%	Relative Risk (RR) of Death	*X* ^2^	Df	*p* Value
AP pre-COVID-19 vs. AP during-COVID-19	3.54 (1.06–11.77)	3.00 (1.03–8.74)	4.667	1	0.031 *
AP pre-COVID-19 vs. AP with COVID-19	9.75 (2.75–34.46)	6.00 (2.12–16.91)	15.441	1	<0.001 *
AP during-COVID-19 vs. AP with COVID-19	2.75 (1.02–7.35)	2.00 (1.03–3.86)	4.200	1	0.040 *

* *p* < 0.05—statistically significant.

## Data Availability

Not applicable.
